# Diets Based on Virgin Olive Oil or Fish Oil but Not on Sunflower Oil Prevent Age-Related Alveolar Bone Resorption by Mitochondrial-Related Mechanisms

**DOI:** 10.1371/journal.pone.0074234

**Published:** 2013-09-16

**Authors:** Pedro Bullon, Maurizio Battino, Alfonso Varela-Lopez, Patricia Perez-Lopez, Sergio Granados-Principal, Maria C. Ramirez-Tortosa, Julio J. Ochoa, Mario D. Cordero, Adrian Gonzalez-Alonso, César L. Ramirez-Tortosa, Corrado Rubini, Antonio Zizzi, José L. Quiles

**Affiliations:** 1 Department of Periodontology, Dental School, University of Sevilla, Sevilla, Spain; 2 Dipartimento di Scienze Cliniche Specialistiche ed Odontostomatologiche, Università Politecnica delle Marche, Ancona, Italia; 3 Institute of Nutrition and Food Technology “José Mataix Verdú”, Department of Physiology, University of Granada, Granada, Spain; 4 Institute of Nutrition and Food Technology “José Mataix Verdú”, Department of Biochemistry and Molecular Biology II, University of Granada, Granada, Spain; 5 Departamento de Citología e Histología Normal y Patológica, Universidad de Sevilla, Sevilla, Spain; 6 Department of Pathology, Complejo Hospitalario de Jaén, Jaén, Spain; 7 Dipartimento di Scienze Biomediche e Sanità Pubblica, Università Politecnica delle Marche, Ancona, Italia; University of Salento, Italy

## Abstract

**Background/Objectives:**

Aging enhances frequency of chronic diseases like cardiovascular diseases or periodontitis. Here we reproduced an age-dependent model of the periodontium, a fully physiological approach to periodontal conditions, to evaluate the impact of dietary fat type on gingival tissue of young (6 months old) and old (24 months old) rats.

**Methods/Findings:**

Animals were fed life-long on diets based on monounsaturated fatty acids (MUFA) as virgin olive oil, n-6 polyunsaturated fatty acids (n-6PUFA), as sunflower oil, or n-3PUFA, as fish oil. Age-related alveolar bone loss was higher in n-6PUFA fed rats, probably as a consequence of the ablation of the cell capacity to adapt to aging. Gene expression analysis suggests that MUFA or n-3PUFA allowed mitochondria to maintain an adequate turnover through induction of biogenesis, autophagy and the antioxidant systems, and avoiding mitochondrial electron transport system alterations.

**Conclusions:**

The main finding is that the enhanced alveolar bone loss associated to age may be targeted by an appropriate dietary treatment. The mechanisms involved in this phenomenon are related with an ablation of the cell capacity to adapt to aging. Thus, MUFA or n-3PUFA might allow mitochondrial maintaining turnover through biogenesis or autophagy. They might also be able to induce the corresponding antioxidant systems to counteract age-related oxidative stress, and do not inhibit mitochondrial electron transport chain. From the nutritional and clinical point of view, it is noteworthy that the potential treatments to attenuate alveolar bone loss (a feature of periodontal disease) associated to age could be similar to some of the proposed for the prevention and treatment of cardiovascular diseases, a group of pathologies recently associated with age-related periodontitis.

## Introduction

Aging, a common phenomenon to all multicellular organisms, is described as an endogenous and progressive decay in the efficacy of physiological processes after the reproductive phase [1]. Aging is related to some chronic diseases like diabetes, cancer, cardiovascular and neurodegenerative diseases. Periodontitis, also related to age, is a disorder characterized by the breakdown of the tooth-supporting tissues, mainly alveolar bone loss. This condition is due fundamentally to an ecological imbalance between the normal microbial biofilm on teeth and the host tissues [2]. There is increasing evidence linking periodontitis to systemic diseases like atherosclerosis [3]. Metabolic syndrome is a clinical entity that encompasses several risk factors for cardiovascular diseases and that has been related with periodontitis; with oxidative stress being proposed as a potential common link to explain this relationship [4,5].

The development of interventions to retard the aging process, focused on extending maximum life span and/or retarding the broad spectrum of age-associated biological changes, is of great importance. To date, in laboratory rodents, the most successful dietary intervention has been caloric restriction, although in the past few years it has been demonstrated that some antioxidants, as well as interventions in relation to dietary fat, may also be used as dietary anti-aging therapies [6,7]. It has been shown that dietary fat, through changes in membrane-lipid profiles, may help to attenuate some deleterious aspects of aging, such as those related to exacerbated oxidative stress or mitochondrial dysfunction [7-11]. Regarding this, we have previously described how n-6 PUFA-rich diets lead to higher levels of lipid peroxidation and DNA double-strand breaks in rat tissues and blood during aging, compared with the less oxidizable, MUFA-based diets [7,8]. In parallel, MUFA diets lead to less damaged mitochondria during aging than n-6 PUFA diets, as reflected by a lower frequency of mtDNA (mitochondrial DNA) deletions at the liver, heart and brain [7,10,12].

Traditionally, much of the research on diet and periodontal diseases isfocused on only a few nutrients with well-established roles in the formation and maintenance of structural components of oral tissue such as collagen (vitamin C) and bone (calcium), integrity of epithelial tissue (vitamin A), or in promoting the formation of plaque that harbors periodontal pathogens (carbohydrates). More recently, research has expanded to include nutrients shown to attenuate the inflammatory process or to have anti-inflammatory properties, like some n-3 and n-6 PUFA [13]. However, no research has been performed on the putative role of other well-documented healthy fatty acids such as MUFA.

Several studies have been carried on to experimentally induce periodontal disease in rat models. Generally these models are based on infection induced by pathogenic agents to reproduce, in a short period of time, the features of the disease. However, all these studies do not take in account the impaired host response and the alveolar bone conditions related to age. The present study reproduces an age-dependent model of the periodontium, which is a fully physiological approach to the periodontal conditions, with the aim (i) of evaluating the impact of the nature of dietary fat on periodontal tissue conditions and (ii) to explore some possible mechanisms involved, other than the well-known aspects directly related to inflammation.

## Materials and Methods

### Ethics Statement

The animals were treated in accordance with the guidelines of the Spanish Society for Laboratory Animals and the experiment was approved by the Ethical Committee of the University of Granada (permit number 20-CEA-2004).

### Chemicals

All the chemical products and solvents, of the highest grade available, were acquired from Sigma (St. Louis, MO, USA) and Merck (Darmstadt, Germany). 

### Animals and Diets

Seventy two male Wistar rats (*Rattus norvegicus*) weighing 80-90g were housed 3 to a cage and maintained in a 12 h light/12h darkness cycle, with free access to food and water. The rats were randomly assigned into three experimental groups and fed from weaning until 24 months of age on a semi-synthetic and isoenergetic diet according to the AIN93 criteria [14] composed of (in g/100g of diet): 14 casein, 46.57 starch, 10 sucrose, 15.5 Dextrose, 4 dietary fat, 5 cellulose, 0.25 choline, 0.18 L-cystine, 1.0 vitamin mixture and 3.5 mineral mixture. The experimental diets differed only in the dietary-fat source (virgin olive, sunflower or fish oils, Table 1). Three groups were established: animals fed on virgin olive oil, sunflower oil or fish oil. Twelve rats per group were killed, respectively, at 6 and 24 months from the start of the experiment. The rats were killed by cervical dislocation followed by decapitation, at the same time of the day, to avoid any circadian fluctuation. Blood was collected in EDTA-coated tubes, and plasma was centrifuged at 1750 x *g* for 10 min. After exsanguination, gingival tissue was excised from around the maxillary molars for use in quantitative real-time PCR to assess mRNA expression of selected genes and other molecules of interest.

**Table 1:** Fatty acids profile of experimental dietary fats (g/100g).

**Fatty acid or index**	**Virgin olive**	**Sunflower**	**Fish oil**
C14:0	0.0	0.1	7.2
C16:0	8.3	6.4	17.1
C16:1n-9	1.1	0.1	9.6
C18:0	3.2	4.7	2.7
C18:1n-9	77.7	24.2	15.1
C18:2n-6	3.2	62.8	2.8
C20:3n-6	0.1	0.9	0.1
C20:4n-6	0.0	0.0	2.1
C20:5n-3	0.2	0.1	18.6
C24:0	0.0	0.1	0.3
C24:1n-9	0.0	0.0	0.9
C22:6n-3	0.0	0.0	10.5
Total saturated fatty acids	12.6	11.5	30.5
Total monounsaturated fatty acids	83.7	24.4	30.1
Total n-6 polyunsaturated fatty acids	3.3	63.7	8.2
Total n-3 polyunsaturated fatty acids	0.4	0.4	31.3
Total polyunsaturated fatty acids	3.7	64.1	39.4

### Alveolar Bone Level and Gingival Histology

The left side of the mandible was defleshed by immersion in 10% hydrogen peroxide (3 to 4 days at room temperature). The soft tissues were carefully removed, and the mandible was stained with methylene blue for good visual distinction between the tooth and bone. Measurements were made at two points at mesial and distal of second premolar and first molar to quantify crestal bone level. The mandibles were photographed using an inverted microscope at x10 (Olympus SZ61®). The captured image was also analyzed using ImageJ software (NIH USA), and the mean crestal bone level was calculated for each group. The mean value for each tooth was calculated. Mean value obtained at 6 months was considered as baseline and the changes in bone level were calculated with 24 months data.

For histologic analysis, samples taken from molar gingival mucosa were fixed in buffered formalin and paraffin-embedded. Tissue sections were cut with a microtome and stained with haematoxylin and eosin for histological examination. Measures in the connective tissue were thickness as the distance between epithelium and bone; number of total vessels and of vessels with endothelial activation counted in 5 consecutive high power fields; degree of fibrosis expressed with a scoring system (grade 1=less than 2 thick collagen bundles, 2=between 2 and 5 thick collagen bundles, 3=more than 5 thick collagen bundles); cellularity as number of fibroblast-like cells per high power field; degree of inflammation (grade 1=absent, 2=mild, 3=moderate).

### RT-PCR

Gingival tissues were preserved in RNAlater at -80°C. Total RNA was extracted using RNeasy Lipid Tissue Mini Kit (Qiagen) following manufacturer’s condition. The quantity and purity of the RNA were determined from the absorbance at 260/280 nm. 20ng of total RNA was reverse-transcribed into cDNA using Multiscribe enzyme (Applied Biosystems) in accordance with the manufacturer’s protocol. Abi 7900 Real-Time PCR system and real time PCR kit (TaqMan® Gene Expression Assays, Applied Biosystems) were employed based on the manufacturer’s instruction. GADPH was used as an internal control. The probes of studied genes and GADPH are shown in Table 2. PCR thermal cycling were development in three steps. Step 1 at 50°C lasting 2 minutes. Step 2 at 95°C lasting 10 minutes and a final step with 40 cycles at 95°C lasting 15 seconds and at 60°C lasting 1 minute.

**Table 2:** Assay ID of probes used in TaqMan® Gene Expression Assays (Applied Biosystems).

	Assay ID	Reference sequence
IL1b	Rn99999009_m1	NM_031512.2
Il6	Rn99999011_m1	NM_012589.1
IL8	Rn00567841_m1	NM_017183.1
Tnfα	Rn99999017_m1	NM_012675.3
OPG	Rn00563499_m1	NM_012870.2
RANKL	Rn00589289_m1	NM_057149.1
Bcl2	Rn99999125_m1	NM_001033672.1
Bad	Rn00575519_m1	NM_022698.1
LC3	Rn01536227_g1	NM_199500.1
Atg5	Rn01767063_m1	NM_001014250.1
Tfam	Rn00580051_m1	NM_031326.1
MT-ND1	Rn03296764_s1	
MT-ND4	Rn03296781_s1	
PGC1	Rn01511922_m1	NM_133284.2
Sirt1	Rn00490758_m1	NM_001153061.1
Ndufs1	Rn01438310_g1	NM_001005550.1
Sod1	Rn00566938_m1	NM_017050.1
Sod2	Rn99999088_g1	NM_017051.2
Nrf2	Rn00477784_m1	NM_010902.3
Gadph	Rn99999916_s1	NM_017008.3

### Multiplexed Beads Immunoassays for Serum OPG and RANKL

OPG and RANKL were measured simultaneously using a high sensitivity human cytokine multiplex immunoassay (Milliplex^TM^ MAP). Assays were run on a Luminex^®^ X-MAP Bio-Plex 200 System Bioanalyzer (Luminex Corp., Austin, TX) according to the kit manufacturer’s instructions.

### Lipid Peroxidation Analysis at the Gums

TBARS (Thiobarbituric acid-reactive substances) levels were determined by a method based on the reaction with thiobarbituric acid at 90–100 °C according to Harma et al. [15]. Shortly, the reaction was performed at 90°C for 15 min pH 2–3. The sample was mixed with two volumes of cold 10% (w/v) trichloroacetic acid to precipitate protein. This precipitate was pelleted by centrifugation and an aliquot of the supernatant was reacted with an equal volume of 0.67% (w/v) TBA in a boiling water bath for 10 min. After cooling, the absorbance was read at 532 nm. Results were expressed as nmol/mL.

### Fatty Acid Profile of Plasma and Dietary Fat Sources

The fatty-acid profile of plasma and dietary fat sources was measured by gas-liquid chromatography following Lepage and Roy method [16] as previously described [9]. Briefly, a gas-liquid Chromatograph model HP-5890 Series II (Hewlett Packard, Palo Alto, CA, USA) equipped with a flame-ionization detector was used to analyze the fatty acids as methyl esters. Chromatography was performed using a 60-m-long capillary column 32 mm id and 20 mm thick impregnated with Sp 2330TM FS (Supelco Inc, Bellefonte, Palo Alto, CA, USA). The injector and the detector were maintained at 250 and 275°C, respectively; nitrogen was used as the carrier gas, and the split ratio was 29:1. Temperature programming (for a total time of 40 min) was as follows: initial temperature, 160°C for 5 min, 6ºC/min to 195°C, 4°C/min to 220°C, 2ºC/min to 230°C, maintained 12 min, 14ºC/min to 160°C.

### Statistical Analysis

Results represent the mean and the standard error of the mean of 6 animals. Variables were checked for normality and homogeneous variance. When a variable was found not to follow normality, it was log-transformed and reanalyzed. To evaluate differences in the means between groups at 6 and 24 months, one-way ANOVA analysis followed by a multiple-comparison test, adjusted by Bonferroni correction, was performed. To test differences between dietary treatments between the two age periods, Student’s t-test were performed. Data were analyzed using the SPSS/PC statistical software package (SPSS for Windows, 15.0.1, 2006, SPSS Inc., Chicago, IL, USA).

## Results

### Body Weight and Food Intake

The body weights of the animals were similar for all groups at six months (309 ± 9 g for virgin olive oil group, 301 ± 11 g for sunflower oil group and 315 ± 13 g for fish oil group). At twenty four months, fish oil groups showed higher body weight (604 ± 23 g) than virgin olive oil group (534±52g) and sunflower oil group (508 ± 19 g). No differences concerning food intake were found between groups or in relation to age. 

### Alveolar Bone Level and Gingival Histology

**Figure 1:**
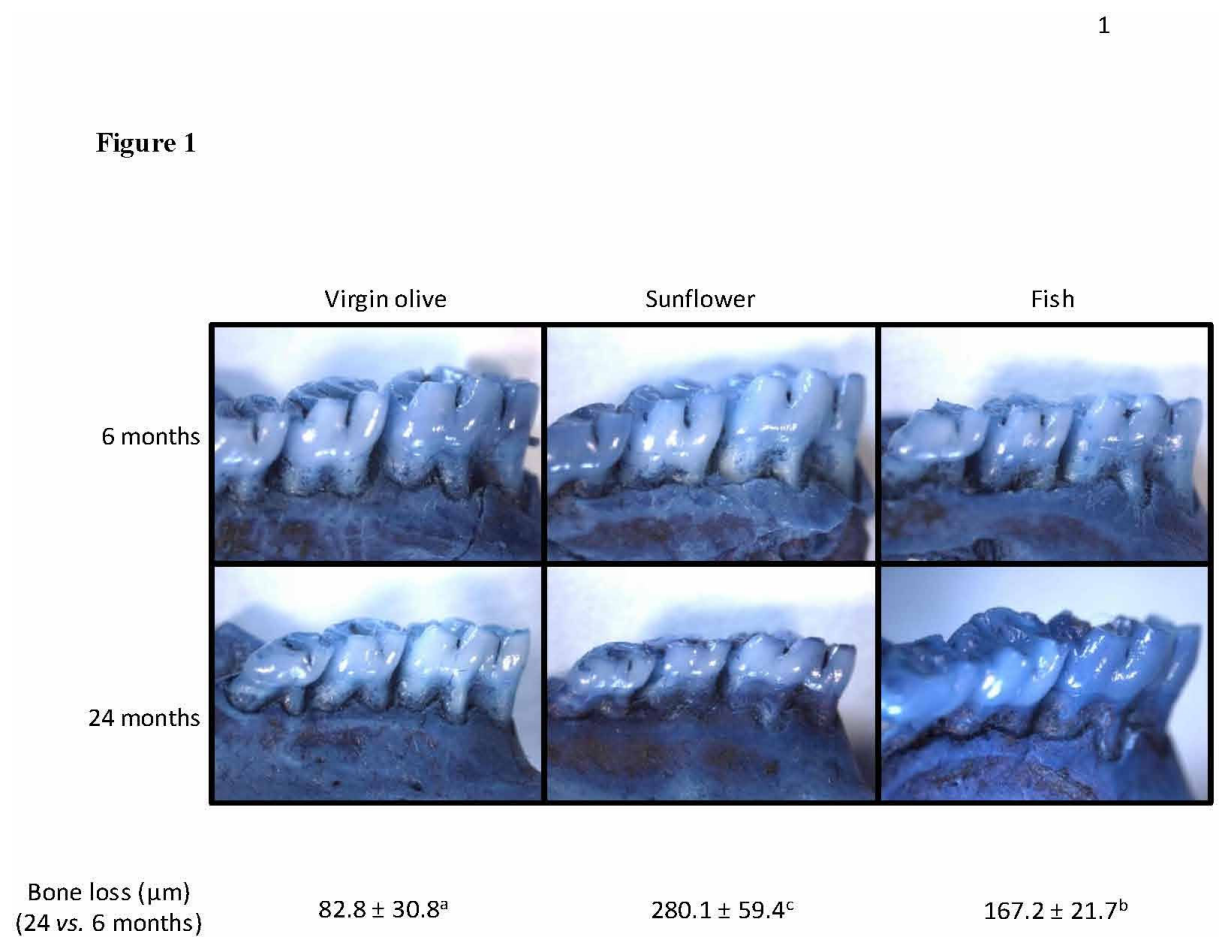
Effects of dietary fat type on alveolar bone loss in the mesial and distal roots of the second premolar and the first molar of young (6 months old) and old (24 months old) rats. Results are presented as mean ± EEM of bone loss between 6 and 24 months. Lower-case letters, when different, represent statistically significant differences (*P*< 0.05) between the three dietary treatments. The figure also shows dental cervical area after stained with methylene blue.

**Figure 2:**
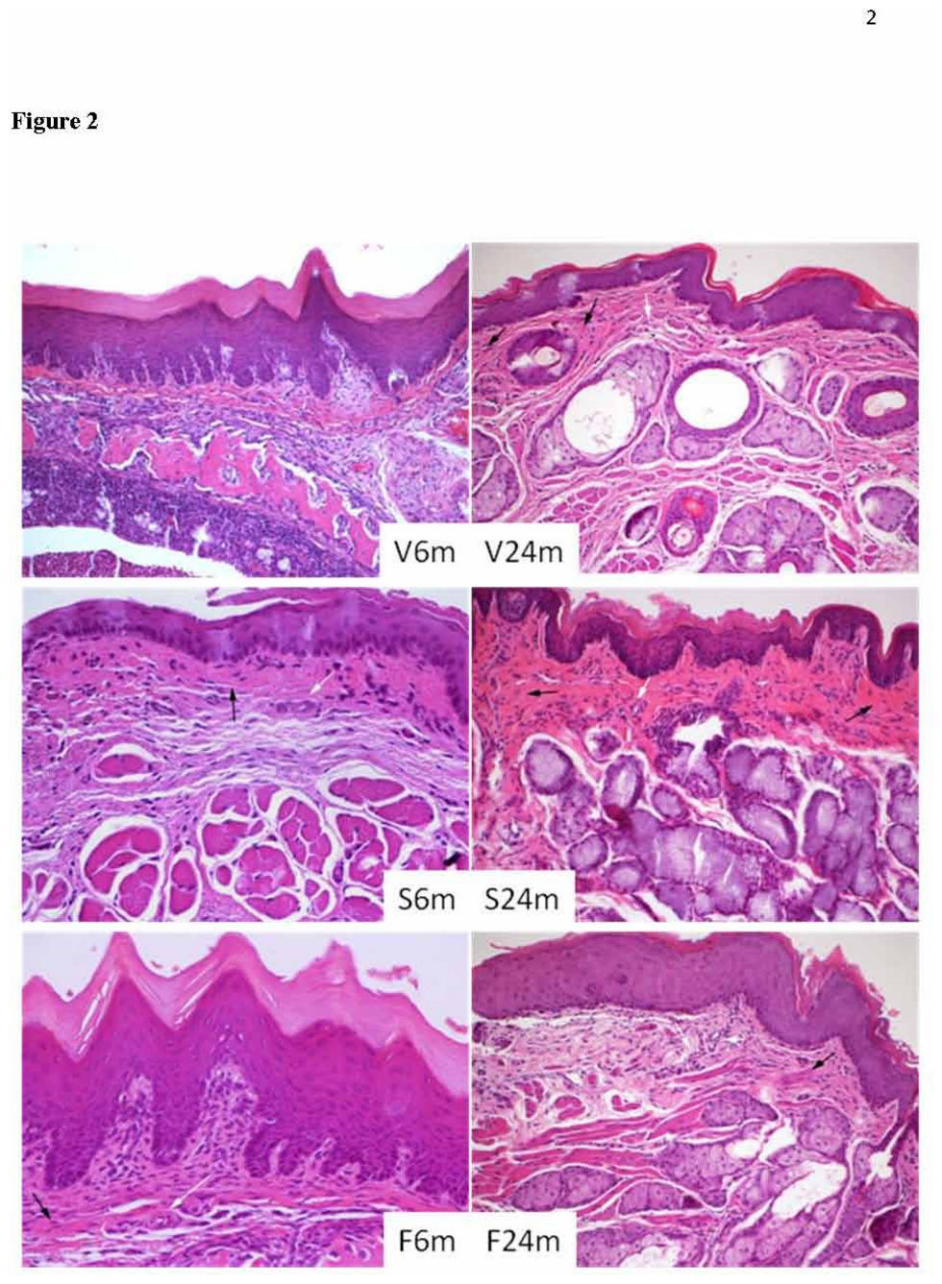
Histological features of gingival tissue in young (6 months old, left column) and old (24 months old, right column) rats fed on virgin olive (V), sunflower (S) or fish (F) oils. White arrows show areas with activated endothelial vessels. Black arrows show areas with fibrosis.

Animals fed on sunflower oil showed the significantly highest bone loss, followed by those fed on fish oil (Figure 1). Concerning histopathological findings, results are reported in table 3 and Figure 2. The groups did not differ significantly for thickness. At 24 months, the number of vessels was lower for all groups, compared to 6 months, particularly in the sunflower group. The endothelial cells tended to be activated at 24 months. At 24 months, only the sunflower group showed a high degree of fibrosis and a moderate degree of inflammation; both sunflower and fish showed a reduction of cellularity.

**Table 3:** Histological findings in rat tissue sections stained with haematoxylin and eosin.

	6 months			24 months		
	Virgin olive	Sunflower	Fish	Virgin olive	Sunflower	Fish
Thickness (mm)	0.25	0.23	0.21	0.24	0.22	0.23
Vessel number	9.8±1.2	9.6±1.2	10.2±1.4	8.9±1.1	8.3±1.1	9.2±1.3
Endothelial activation (vase x 5HPF)	1	2	2	2	4	4
Fibrosis	+/-	+	+	+/-	++	+
Cellularity (cells x HPF)	<20	<20	<20	<20	<10	<10
Inflammation	-	-	+/-	+/-	+	+/-

### Plasma Fatty Acid Profile

Table 4 shows the plasma lipid profile of rats. For saturated fatty acids, the highest proportion for C14: 0 and C16: 0 were found for fish oil fed animals, which showed the lowest proportion for C18: 0. For MUFA, virgin olive oil fed rats showed the highest proportion for C18: 1n-9 and total MUFA index. Individual and total n-6PUFA index were the highest for sunflower oil group. For n-3 PUFA, fish oil fed animals reported the highest proportions.

**Table 4:** Plasma fatty acids in rats fed on different dietary fat sources (g/100g).

Fatty acid or index	Virgin olive	Sunflower	Fish
C14:0	0.7±0.1^a^	0.6±0.1^a^	1.1±0.1^b^
C16:0	20.9±1.3^a^	20.8±0.5^a^	25.8±1.3^b^
C16:1n-9	5.5±0.5^b^	3.6±0.6^a^	6.7±0.3^c^
C18:0	12.4±0.5^b^	12.8±0.5^b^	9.9±0.5^a^
C18:1n-9	26.0±5.3^b^	10.5±5.3^a^	11.9±3.7^a^
C18:2n-6	7.3±0.4^b^	14.7±2.2^c^	2.3±0.7^a^
C20:3n-6	0.3±0.1^ab^	0.4±0.1^b^	0.2±0.1^a^
C20:4n-6	6.7±0.2^a^	15.7±1.9^b^	5.8±0.4^a^
C20:5n-3	0.2±0.1^a^	0.1±0.0^a^	8.4±1.1^b^
C24:0	0.8±0.1^a^	1.6±0.4^b^	0.8±0.3^a^
C24:1n-9	0.9±0.2^a^	1.1±0.3^ab^	1.5±0.2^b^
C22:6n-3	2.0±0.2^b^	0.6±0.3^a^	9.0±0.6^c^
Total saturated fatty acids	37.2±1.5^a^	38.5±1.1^a^	40.6±1.7^a^
Total monounsaturated fatty acids	40.6±1.8^b^	27.9±3.3^a^	31.6±2.4^a^
Total n-6 polyunsaturated fatty acids	19.8±0.7^b^	32.2±2.7^c^	9.5±0.4^a^
Total n-3 polyunsaturated fatty acids	2.4±0.1^b^	1.4±0.3^a^	18.3±1.2^c^
Total polyunsaturated fatty acids	22.2±0.7^a^	33.6±2.5^c^	27.8±1.3^b^

Results show means ± EEM. Lower-case letters, when different, represent statistically significant differences (*P*<0.05) between the three dietary treatments.

### Interleukin-Related mRNA Levels

**Figure 3:**
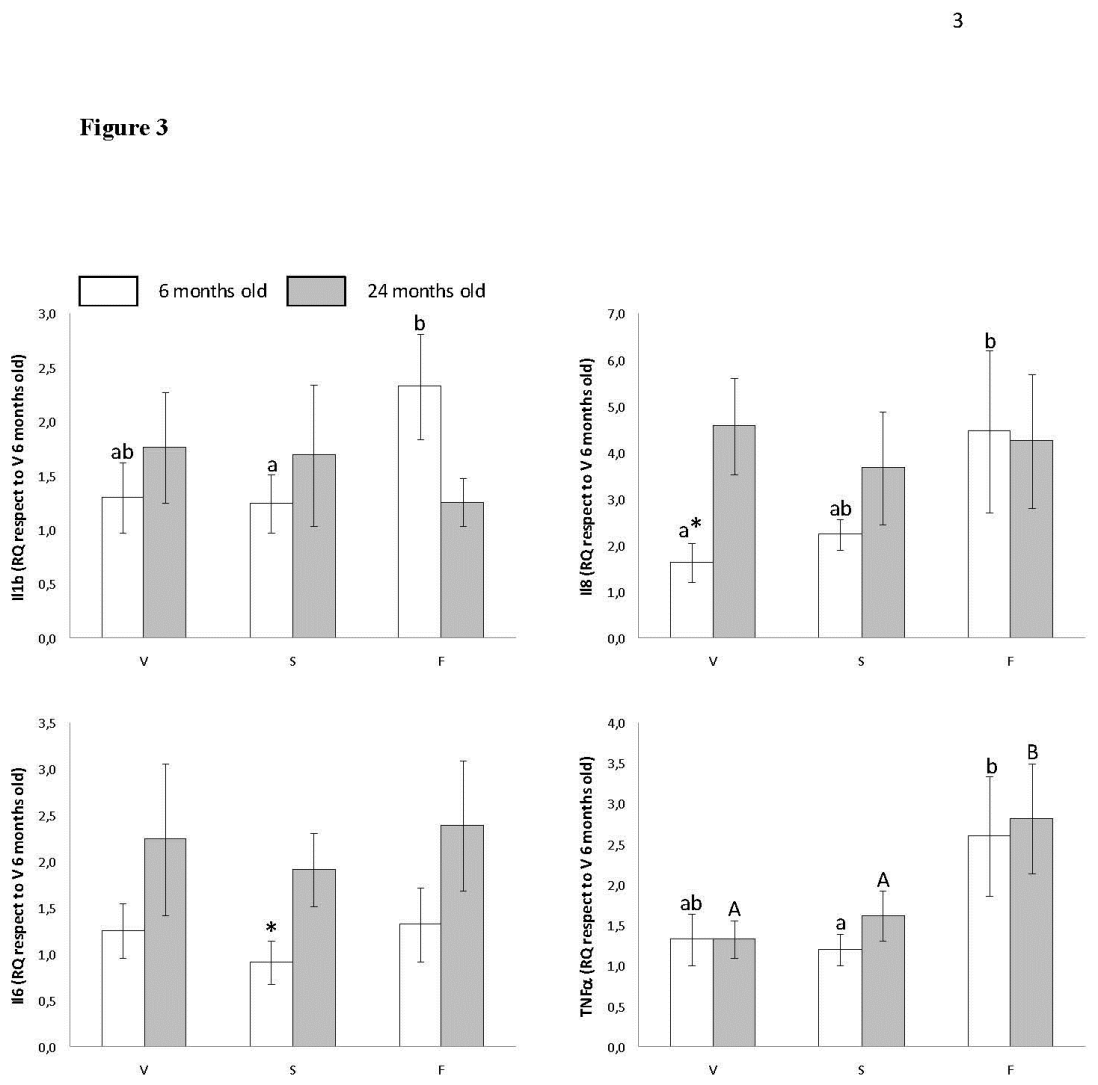
Effects of dietary fat type on Interleukin-related mRNA levels in gingival tissue of young (6 months old) and old (24 months old) rats fed on virgin olive (V), sunflower (S) or fish (F) oils. Results are presented as mean ± EEM. Asterisk (*) means a statistically significant difference between the same dietary treatment for rats aged 6 and 24 months. Lower-case letters, when different, represent statistically significant differences (*P*< 0.05) between the three dietary treatments at 6 months. Upper-case letters, when different, represent statistically significant differences (*P*< 0.05) between the three dietary treatments at 24 months.

Figure 3 shows the relative quantity (RQ) of Il1b, Il8, Il6 and TNFα in gum mRNA. Concerning Il1b, changes were found only between treatments at six months of age, showing that fish oil fed animals had a higher RQ than sunflower oil fed animals. Similar changes were also found for Il8. Also differences between six and twenty four months were found for virgin olive oil fed animals, showing old animals with a higher RQ. Concerning Il6, changes between six and twenty four months were found only for sunflower oil group, with higher RQ at twenty four months. TNFα RQ showed no age-related differences. Animals fed on fish oil presented the highest RQ both at six and at twenty four months.

### mRNA and Circulating Levels of Bone Resorption Markers

**Figure 4:**
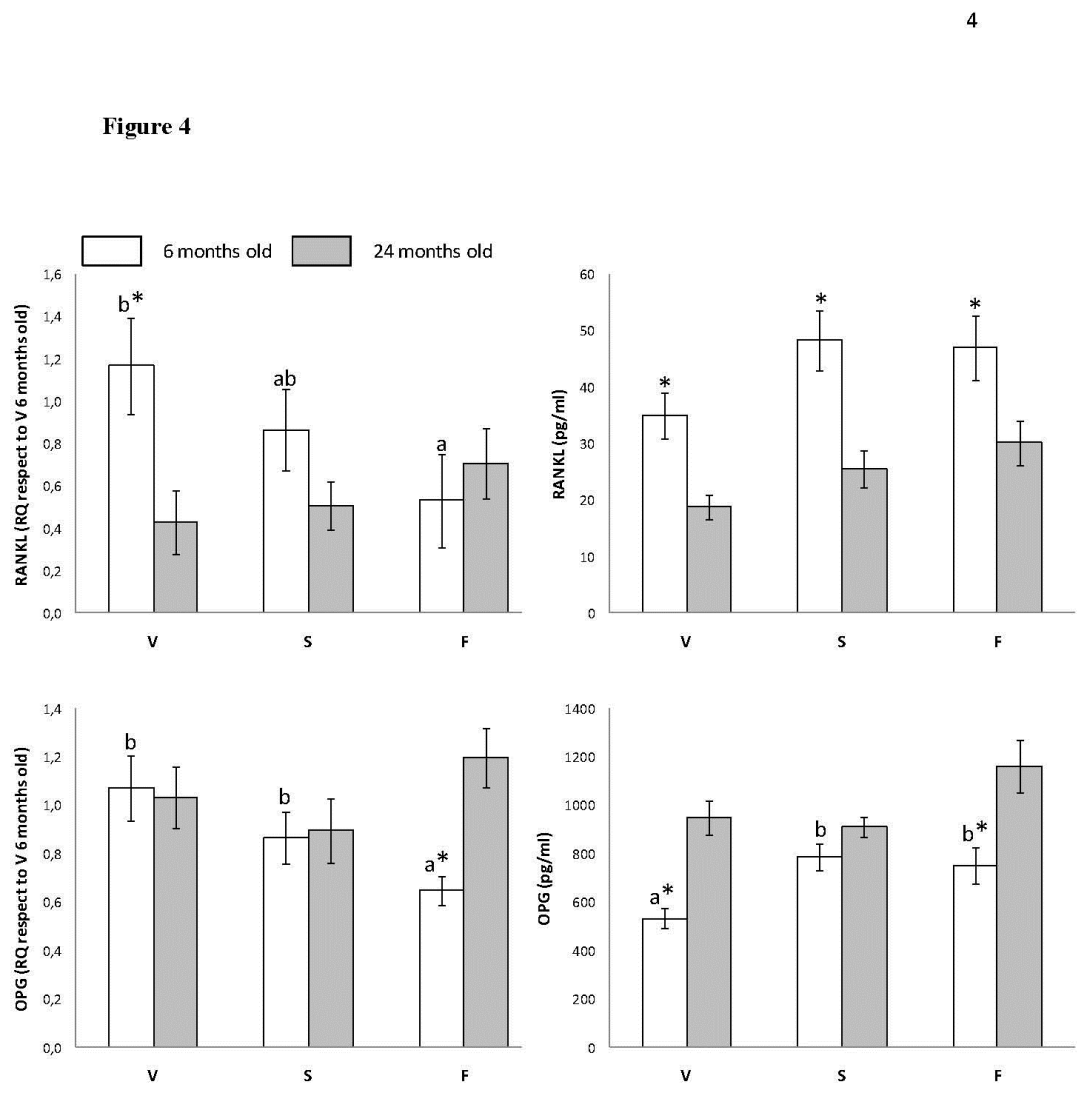
Effects of dietary fat type on gingival mRNA and circulating levels of bone resorption markers (RANKL and OPG) of young (6 months old) and old (24 months old) rats fed on virgin olive (V), sunflower (S) or fish (F) oils. Results are presented as mean ± EEM. Asterisk (*) means a statistically significant difference between the same dietary treatment for rats aged 6 and 24 months. Lower-case letters, when different, represent statistically significant differences (*P*< 0.05) between the three dietary treatments at 6 months. Upper-case letters, when different, represent statistically significant differences (*P*< 0.05) between the three dietary treatments at 24 months.

Figure 4 shows circulating levels and RQ in gum mRNA for RANKL and OPG. Concerning RANKL, RQ was the highest for virgin olive oil fed animals at 6 months. Age-related differences were found only for virgin olive oil group, showing higher values at six months. Concerning plasma RANKL, differences were found only associated to age, with animals at six months showing higher levels, for all dietary treatment, than those at twenty four months. For OPG RQ mRNA, the lowest value was found for fish oil fed animals at six months. Age-related changes were found only for fish oil groups, with animals at twenty four months showing higher values than those at six months. Plasma OPG reported at six months that animals fed on virgin olive oil showed the lowest concentration. Virgin olive oil and fish oil fed rats reported higher concentrations at twenty four than at 6 months.

### Apoptosis, Autophagy, Biogenesis, Mitochondrial Electron Transport Chain and Antioxidant Enzymes-Related Genes

RQ mRNA for genes related to apoptosis, autophagy, biogenesis, mitochondrial electron transport chain and antioxidant enzymes are presented in table 5. Concerning apoptosis, RQ for Bad, age related differences were found for virgin olive- and fish oil fed groups, with old animals showing higher values than those at six months of age. Concerning Bax, differences were found only between six and twenty four months for virgin olive oil, with the aged group showing the highest RQ. RegardingBcl2, RQ at six months was higher for sunflower oil fed animals compared with fish oil fed animals. Age-related changes were found for virgin olive- and fish- oil fed animals, always with higher values for old animals. In relation to autophagy, for Atg5, differences between young and old animals were found for virgin olive- and fish- oil groups, with old animals showing higher values. In relation to LC3, at six months, the highest RQ was found for sunflower oil group. At twenty four months, the highest RQ was found for virgin olive oil fed animals. Differences concerning age were found for virgin olive oil and fish oil groups, with old animals showing higher values than the young animals.

**Table 5:** Effects of dietary fat type on gingival mRNA levels (RQ) of genes related to apoptosis (Bad, Bax and Bcl2), autophagy (Atg5 and LC3), biogenesis (Tfam, PGC1 and Sirt1), mitochondrial electron transport chain (MT-ND1, MT-ND4 and Ndfus1) and antioxidant system (Nrf2, SOD1 and SOD2), as well as gum levels of thiobarbituric acid reactive substances (TBARS, nmol/mg) of young (6 months old) and old (24 months old) rats.

	6 months			24 months		
	Virgin olive	Sunflower	Fish	Virgin olive	Sunflower	Fish
Bad	1.09±0.17*	1.46±0.12	1.11±0,11*	3.29±0.74	2.85±0.09	1.98±0.12
Bax	1.10±0.17*	1.57±0.28	1.11±0,19	2.91±0.76	2.11±0.48	2.12±0.70
Bcl2	1.05±0.13^ab^*	1.42±0.27^b^	0.95±0,04ª*	2.19±0.41	1.93±0.50	2.13±0.41
Atg5	1.06±0.14*	1.33±0.28	0.94±0,09*	1.97±0.45	1.53±0.26	1.58±0.36
LC3	1.07±0.14^ab^*	1.43±0.34^b^	0.89±0,09ª*	2.78±0.53^B^	1.71±0.40^A^	1.68±0.43^A^
Tfam	1.11±0.19*	1.30±0.26	0.82±0,09*	2.99±0.59^B^	1.24±0.26^A^	1.63±0.44^A^
PGC1	1.15±0.23*	1.50±0.33	1.90±0.34*	4.07±0.51^B^	1.91±0.48^A^	3.06±0.19^A^
Sirt1	1.36±0.12*	1.43±0.21	0.87±0.33*	2.95±0.38^B^	1.41±0.21^A^	1.83±0.18^A^
MT-ND1	1.17±0.21*	1.59±0.44	0.89±0,29	3.78±0.80^B^	1.25±0.31^A^	1.74±0.38^A^
MT-ND4	1.14±0.18^b^*	1.50±0.40^b^	0.71±0,10ª*	3.04±0.78^B^	1.09±0.27^A^	1.30±0.26^A^
Ndfus1	1.21±0.26^b^*	1.56±0.40^b^	0.61±0,05ª*	3.39±0.75^B^	1.08±0.28^A^	1.19±0.30^A^
SOD1	1.09±0.16ª*	1.72±0.40^b^	0.87±0,81ª*	2.68±0.65	2.02±0.26	2.26±0.83
SOD2	1.13±0.21^ab^*	1.67±0.42^b^	0.81±0,07ª*	2.73±0.77	2.15±0.68	1.75±0.45
Nrf2	1.07±0.09^a^*	1.61±0.21^b^*	1.05±0.13^a^*	3.00±0.39	2.64±0.36	3.25±0.49
TBARS (nmol/mg)	0.13±0.01*	0.16±0.01	0.14±0.03*	0.09±0.01^B^	0.16±0.02^C^	0.06±0.01^A^

Results show means ± EEM. Asterisk (*) means statistically significant difference between the same dietary treatment for rats aged 6 and 24 months. Lower-case letters, when different, represent statistically significant differences (*P*< 0.05) between the three dietary treatments at 6 months. Upper-case letters, when different, represent statistically significant differences (*P*< 0.05) between the three dietary treatments at 24 months.

Concerning biogenesis, for Tfam RQ at twenty four months, the highest value was found for virgin olive oil fed animals. Virgin olive oil and sunflower oil groups presented higher RQ at twenty four months than at six months. Similar results were found for PGC1 and Sirt1 RQ. Three genes related to complex I from the mitochondrial electron transport chain were analyzed. Two of these genes are from mitochondrial DNA origin (MT-ND1 and MT-ND4) and the other from nuclear origin (Ndufs1). Concerning MT-ND1, at twenty four months virgin olive oil fed animals showed the highest RQ. Differences related to ageing were found for virgin olive oil group, with higher RQ at twenty four months. In relation to MT-ND4 and Ndufs1, similar results were found. At six months the lowest RQ was found for fish oil fed animals. At twenty four months, the highest value was found for virgin olive oil group. Age-related differences were found for virgin olive and fish oil groups, being higher at twenty four months. Regarding the antioxidant enzyme superoxide dismutase (SOD), both cytoplasmic (SOD1) and mitochondrial (SOD2) reported almost similar RQ. Sunflower oil led to the highest RQ at six months. Virgin olive and fish oil presented higher values at twenty four months than at six months. Similar values were found for Nrf2 RQ.

### Lipid Peroxidation at the Gums

Lipid peroxidation at the gums was evaluated through the TBARS assay (table 5). At twenty four months the highest concentration was found for sunflower oil fed animals. Age led to differences for virgin olive and fish oil fed animals, with higher concentrations at six months, compared with twenty four months.

## Discussion

Rats fed on virgin olive oil showed the highest relative concentrations of MUFA, the rats fed on sunflower oil showed the highest relative concentrations of n-6 PUFA and the rats fed on fish oil showed the highest relative concentrations of n-3 PUFA. These finding suggest a proper adaptation of animals to dietary fat [17,18]. In humans, age has been shown to affect the severity and progression of periodontitis, with older adults appearing to carry a disproportionate risk for periodontitis [19,20]. Some other studies have also been carried out in the same way [e.g. 21,22]. In 1994 [22], Burt reported that a loss of periodontal attachment and alveolar bone tended to occur in older patients. Moreover it was proven that aging of the periodontal tissue participates in the development of periodontitis in elderly persons [23]. In rats, Arai et al. [24] stated that the periodontal bone of rats has similar profiles to humans in terms of resorption with aging. These authors suggested that the rat is a good model for the study of periodontal research on elderly humans. Our results showed differences regarding alveolar bone loss with aging depending on the dietary fat. Sunflower oil fed animals reported the highest bone loss, followed by those rats fed on fish oil and finally by animals fed on virgin olive oil. Several studies have investigated the potential effects of different fatty acids, mainly n-3 PUFA, derived from diet or as supplements, on periodontal disease, both in humans [25-30], and in experimental models [31-33]. In humans, almost irrelevant has been the study of MUFA, with one study using olive oil (not virgin olive oil) as placebo [25]. Two studies focused on gingivitis [25,26] and results did not show positive effects for n-3 PUFA treatment, but it should be highlighted that alveolar bone loss never occurs among gingivitis outcomes. However, in general, and despite the varying experimental conditions (patient size, periodontal measures, and others), a protective association of n-3 PUFA on periodontitis was found after treatment or with higher intake of these fatty acids [27,28,30]. In animals, all revised studies focused on n-3 PUFA. Results were different depending on the dietary treatment schedule, duration and on the way of inducing periodontitis. Overall, when periodontitis was induced by injection of bacterial lipopolisaccharide, n-3 PUFA did not lead to reductions in alveolar bone loss [31]. However, when bacterial inoculation was used, n-3 PUFA reduced alveolar bone loss in a significant manner [32,33]. In any case, these studies do not take in account the influence of age and diet in periodontal health.

Our study is based on the development of alveolar bone resorption, based on aging. This model gives us some important data of what happens in humans, more than with those obtained by the simple inoculation of bacteria or bacterial-derived toxins. Nevertheless, and irrespective of the way periodontitis was induced, it is well known that the onset of this disease is due to bacterial infection [5]. Most studies have focused on inflammation, and to a lesser extent, on other mechanistical events like apoptosis. In the present study, we measured circulating levels of several cytokines (data not showed) together with mRNA relative amount of IL1b, IL6, IL8 and TNFα in gingival tissue. Overall, circulating levels of inflammatory cytokines were higher for older groups but not different depending on treatment (data not showed), and something similar happened for mRNA at the gum level. The lack of differences concerning cytokines could be ascribed to the fact that periodontal inflammation was not promoted.

The discovery of the RANK–RANKL–OPG system has brought rapid progress in the understanding of the regulatory mechanisms of osteoclast differentiation and activation exerted by the immune system [34]. Increased RANKL or decreased OPG local expression can cause bone resorption at various sites of the human skeleton. It has been demonstrated that RANKL is up-regulated, whereas OPG is down-regulated in periodontitis, compared to periodontal health, resulting in an increased RANKL/OPG ratio [35]. Here, the RANKL/OPG ratio was higher in old animals compared with the young ones (data not showed) demonstrating that aging is a condition that favors bone loss. We found that mRNA levels of RANKL decreased with aging, as well as circulating levels of this molecule. On the other hand, OPG was higher for old animals, but not for those fed on sunflower oil. Something similar was also true for mRNA levels of OPG at the gingival tissue. It might be expected that aging would lead to increased concentrations and mRNA levels of RANKL and the opposite occurred for OPG, resulting in a loss of bone and thereafter a pro-periodontitis scenario. However, results found in the present study, for virgin olive and fish oil fed animals are in agreement with results from other studies suggesting that OPG acts as a defensive mechanism during aging in order to avoid an excess of bone destruction induced by an excess of RANKL stimulation. In that sense, Wada et al. [36] found that LPS-stimulation of human fibroblasts produced more OPG than RANKL. Benatti et al. [37] found that periodontal ligament cells from aged humans expressed higher mRNA levels of OPG than younger counterparts, meanwhile no changes in RANKL were reported. These authors suggested that the high OPG levels may be a response of the organism in an attempt to counteract the reductions in bone mass that occur throughout adulthood. One interesting finding is that this mechanism would depend on dietary conditions, as suggested by results from the present study.

In order to try to understand mechanisms involving bone resorption in relation to age and dietary fat treatment we analyzed by RT-PCR mRNA abundance for genes related to apoptosis, autophagy, mitochondrial biogenesis, mitochondrial electron transport chain and antioxidant enzymes. Several studies have shown age-related changes in the levels of proteins and factors that regulate apoptosis [e.g. 38]. In gingival tissue, an age-dependent increase has been shown in the number of TUNEL-positive cells in the subepithelial connective tissue [39]. Jarnbring et al. [40] in 2002 found reduced proliferation and increased apoptosis in the most apical part of the pocket in patients with periodontitis, indicating a net loss of keratinocytes in this area. In the present study, aging led to higher values for Bad and Bax, which are pro-apoptotic molecules, but also for the antiapoptotic Bcl2, only for two of the dietary treatments, virgin olive and fish oil, but not for sunflower oil treated animals. Autophagy may occur as a targeted process driven to eliminate damaged mitochondria (mitophagy) and also has important roles in the cellular adaptation to stress, innate immune responses, and as a quality-control mechanism. Again, we found higher values with aging for RQ mRNA in LC3 and ATG5 genes only in animals fed on virgin olive and fish oil groups. Tfam, a marker of the biogenesis process, implicated in the natural turnover of mitochondria [41] reported, as for apoptosis and autophagy-related genes, no differences concerning to the aging process in animals fed on sunflower oil, although animals fed on virgin olive and fish oil led to higher values for aged animals. The other two markers of apoptosis, namely PGC1 and Sirt1, reinforced Tfam observation.

The mitochondrial theory of aging [42] postulates that cellular aging is the product of mutations in the mitochondrial DNA (mtDNA) genome as a result of oxidative damage. As a result of accumulated damage to mtDNA, the mitochondrial blueprints are markedly altered, thus perpetuating the production of aberrant ETC (electron transfer chain) components [43]. According to that, we investigated elements from the mitochondrial ETC together with elements of the antioxidant system and markers of oxidation. In all situations, studied molecules reported similar results than those found for the previously discussed markers. If MDA levels at 24 months are considered, the highest values were found for those animals with the highest n-6PUFA percentage, i.e. animals fed on sunflower oil. It is well know, that despite fish oil having higher total PUFA than sunflower oil, the fact that most of them come from n-3PUFA, which are endowed with anti-inflammatory properties, lead *in vivo* to lower values of lipid peroxidation than seed oils, more rich in n-6PUFA, with high pro-inflammatory capacity. That, together with a potentially different induction of detoxifying enzymes might explain the only apparently contradictory finding regarding MDA levels and fatty acid composition. Something similar may be applied for 6 months old animals, although at this age differences are not significant concerning MDA levels with respect to sunflower oil fed animals. Overall, results from the present study reinforce other recent reports in which the role of virgin olive oil during aging has been highlighted in comparison with sunflower oil. Thus, Santos-González et al. [44] reported how feeding animals on olive oil for a period of 24 months led to a markedly different plasma proteome when compared with sunflower fed animals. In particular, animals fed on virgin olive oil led to a better modulation of inflammation, homeostasis, oxidative stress, and cardiovascular risk during aging.

To summarize, it seems that alveolar bone loss associated to age may be conditioned by dietary fat. In the present study we demonstrated that n-6PUFA are responsible for a greater alveolar bone loss associated to age. The mechanisms involved in this phenomenon are related with an ablation of the cell capacity to adapt to aging. Thus, MUFA or n-3PUFA might allow mitochondrial maintaining turnover through biogenesis or autophagy. They might are also able to induce the corresponding antioxidant systems to counteract age-related oxidative stress, and do not inhibit mtETC machinery. From the nutritional and clinical point of view, the interest of the present study resides in the finding that the excess of alveolar bone loss (a feature of periodontal diseases) associated to age may be targeted by an appropriate dietary treatment. Interesting, some of the potential treatments could be similar to those proposed for the prevention and treatment of some cardiovascular diseases, a group of pathologies recently associated with age-related periodontitis.

## References

[1] Barja G (2002) Rate of generation of oxidative stress-related damage and animal longevity. Free Radic Biol Med 33: 1167-1172. doi:10.1016/S0891-5849(02)00910-3. PubMed: 12398924. 12398924 10.1016/s0891-5849(02)00910-3

[2] Newman HN (1974) Diet, attrition, plaque and dental disease. Br Dent J 136: 491-497. doi:10.1038/sj.bdj.4803220. PubMed: 4531943. 4531943 10.1038/sj.bdj.4803220

[3] Lockhart PB, Bolger AF, Papapanou PN, Osinbowale O, Trevisan M et al. (2012) Periodontal disease and atherosclerotic vascular disease: does the evidence support an independent association?: a scientific statement from the American Heart Association. Circulation 125: 2520-2544. doi:10.1161/CIR.0b013e31825719f3. PubMed: 22514251. 22514251 10.1161/CIR.0b013e31825719f3

[4] Bullon P, Cordero MD, Quiles JL, Morillo JM, Ramirez-Tortosa C et al. (2011) Mitochondrial dysfunction promoted by *Porphyromonas* *gingivalis* lipopolysaccharide as a possible link between cardiovascular disease and periodontitis. Free Radic Biol Med 50: 1336-1343. doi:10.1016/j.freeradbiomed.2011.02.018. PubMed: 21354301. 21354301 10.1016/j.freeradbiomed.2011.02.018

[5] Bullon P, Morillo JM, Ramirez-Tortosa MC, Quiles JL, Newman HN et al. (2009) Metabolic Syndrome and Periodontitis: Is Oxidative Stress a Common Link? J Dent Res 88: 503-518. doi:10.1177/0022034509337479. PubMed: 19587154. 19587154 10.1177/0022034509337479

[6] Armeni T, Principato G, Quiles JL, Pieri C, Bompadre S et al. (2003) Mitochondrial dysfunctions during aging: vitamin E deficiency or caloric restriction--two different ways of modulating stress. J Bioenerg Biomembr 35: 181-191. doi:10.1023/A:1023754305218. PubMed: 12887016. 12887016 10.1023/a:1023754305218

[7] Quiles JL, Ochoa JJ, Ramirez-Tortosa MC, Huertas JR, Mataix J (2006) Age-related mitochondrial DNA deletion in rat liver depends on dietary fat unsaturation. J Gerontol A Biol Sci Med Sci 61: 107-114. doi:10.1093/gerona/61.2.107. PubMed: 16510854. 16510854 10.1093/gerona/61.2.107

[8] Quiles JL, Martínez E, Ibáñez S, Ochoa JJ, Martín Y et al. (2002) Ageing-related tissue-specific alterations in mitochondrial composition and function are modulated by dietary fat type in the rat. J Bioenerg Biomembr 34: 517-524. doi:10.1023/A:1022530512096. PubMed: 12678443. 12678443 10.1023/a:1022530512096

[9] Quiles JL, Ochoa JJ, Ramirez-Tortosa C, Battino M, Huertas JR et al. (2004) Dietary fat type (virgin olive vs. sunflower oils) affects age-related changes in DNA double-strand-breaks, antioxidant capacity and blood lipids in rats. Exp Gerontol 39: 1189-1198. doi:10.1016/j.exger.2004.05.002. PubMed: 15288693. 15288693 10.1016/j.exger.2004.05.002

[10] Quiles JL, Pamplona R, Ramirez-Tortosa MC, Naudí A, Portero-Otin M et al. (2010) Coenzyme Q addition to an n-6 PUFA-rich diet resembles benefits on age-related mitochondrial DNA deletion and oxidative stress of a MUFA-rich diet in rat heart. Mech Ageing Dev 131: 38-47. doi:10.1016/j.mad.2009.11.004. PubMed: 19948181. 19948181 10.1016/j.mad.2009.11.004

[11] Ochoa JJ, Quiles JL, Ibáñez S, Martínez E, López-Frías M, Huertas JR et al. (2003) Aging-related oxidative stress depends on dietary lipid source in rat postmitotic tissues. J Bioenerg Biomembr 35: 267-275. doi:10.1023/A:1024615816839. PubMed: 13678277. 13678277 10.1023/a:1024615816839

[12] Ochoa JJ, Pamplona R, Ramirez-Tortosa MC, Granados-Principal S, Perez-Lopez P, Naudí A et al. (2011) Age-related changes in brain mitochondrial DNA deletion and oxidative stress are differentially modulated by dietary fat type and coenzyme Q10. Free Radic Biol Med 50: 1053–1064. doi:10.1016/j.freeradbiomed.2011.02.004. PubMed: 21335087. 21335087 10.1016/j.freeradbiomed.2011.02.004

[13] Kaye EK (2010) n-3 Fatty Acid Intake and Periodontal Disease. J Am Diet Assoc 110: 1650-1652. doi:10.1016/j.jada.2010.08.017. PubMed: 21034877. 21034877 10.1016/j.jada.2010.08.017

[14] Reeves PG (1997) Components of the AIN-93 diets as improvements in the AIN-76A diet. J Nutr 127: 838-841. PubMed: 9164249. 10.1093/jn/127.5.838S 9164249

[15] Harma M, Harma M, Kocyigit A (2004) Comparison of protein carbonyl and total plasmathiol concentrations in patients with complete hydatidiform mole with those in healthy pregnant women. Acta Obstet Gynecol Scand 83: 857–860. doi:10.1080/j.0001-6349.2004.00608.x. PubMed: 15315598. 15315598 10.1111/j.0001-6349.2004.00608.x

[16] Lepage G, Roy CC (1986) Direct trans-esterification of all classes of lipids in one-step reaction. J Lipid Res 27: 114-120. PubMed: 3958609. 3958609

[17] López-Miranda J, Pérez-Jiménez F, Ros E, De Caterina R, Badimón L, Covas MI et al. (2010) Olive oil and health: summary of the II international conference on olive oil and health consensus report, Jaén and Córdoba (Spain) 2008. Nutr Metab Cardiovasc Dis 20: 284-294. doi:10.1016/j.numecd.2009.12.007. PubMed: 20303720. 20303720 10.1016/j.numecd.2009.12.007

[18] Uauy R (2009) Dietary fat quality for optimal health and well-being: overview of recommendations. Ann Nutr Metab 54: 2-7. doi:10.1159/000220821. PubMed: 19641344. 19641344 10.1159/000220821

[19] Dietrich T, Jimenez M, Kaye EAK, Vokonas PS, Garcia RI (2008) Age-dependent associations between chronic periodontitis/edentulism and risk of coronary heart disease. Circulation 117: 1668–1674. doi:10.1161/CIRCULATIONAHA.107.711507. PubMed: 18362228. 18362228 10.1161/CIRCULATIONAHA.107.711507 PMC2582144

[20] Nesbitt MJ, Reynolds MA, Shiau H, Choe K, Simonsick EM, Ferrucci L (2010) Association of periodontitis and metabolic syndrome in the Baltimore Longitudinal Study of Aging. Aging Clin Exp Res 22: 238–242. PubMed: 20634647. 20634647 10.1007/bf03324802 PMC2929594

[21] Van der Velden U (1991) The onset age of periodontal destruction. J Clin Periodontol 18: 380–383. doi:10.1111/j.1600-051X.1991.tb02304.x. PubMed: 1890216. 1890216 10.1111/j.1600-051x.1991.tb02304.x

[22] Burt BA (1994) Periodontitis and aging: reviewing recent evidence. J Am Dent Assoc 125: 273–279. PubMed: 8157839. 8157839 10.14219/jada.archive.1994.0034

[23] Okamura H, Yamaguchi M, Abiko Y (1999) Enhancement of lipopolysaccharide-stimulated PGE2 and IL-1β production in gingival fibroblast cells from old rats. Exp Gerontol 34: 379–392. doi:10.1016/S0531-5565(99)00006-6. PubMed: 10433392. 10433392 10.1016/s0531-5565(99)00006-6

[24] Arai K, Tanaka S, Yamamoto-Sawamura T, Sone K, Miyaishi O, Sumi Y (2005) Aging changes in the periodontal bone of F344/N rat. Arch Gerontol Geriatr 40: 225–229. doi:10.1016/j.archger.2004.08.005. PubMed: 15814156. 15814156 10.1016/j.archger.2004.08.005

[25] Campan P, Planchand PO, Duran D (1997) Pilot study on n-3 polyunsaturated fatty acids in the treatment of human experimental gingivitis. J Clin Periodontol 24: 907-913. doi:10.1111/j.1600-051X.1997.tb01210.x. PubMed: 9442428. 9442428 10.1111/j.1600-051x.1997.tb01210.x

[26] Eberhard J, Heilmann F, Açil Y, Albers KH, Jepsen S (2002) Local application of n-3 or n-6 polyunsaturated fatty acids in thetreatment of human experimental gingivitis. J Clin Periodontol 29: 364–369. doi:10.1034/j.1600-051X.2002.290413.x. PubMed: 11966935. 11966935 10.1034/j.1600-051x.2002.290413.x

[27] Rosenstein ED, Kushner LJ, Kramer N, Kazandjian G (2003) Pilot study of dietary fatty acid supplementation in the treatment of adult periodontitis. Prostaglandins Leukot Essent Fatty Acids 68: 213–218. doi:10.1016/S0952-3278(02)00272-7. PubMed: 12591005. 12591005 10.1016/s0952-3278(02)00272-7

[28] El-Sharkawy H, Aboelsaad N, Eliwa M, Darweesh M, Alshahat M et al. (2010) Adjunctive Treatment of Chronic Periodontitis With Daily Dietary Supplementation With Omega-3 Fatty Acids and Low-Dose Aspirin. J Periodontol 81: 1635-1643. doi:10.1902/jop.2010.090628. PubMed: 20572767. 20572767 10.1902/jop.2010.090628

[29] Iwasaki M, Yoshihara A, Moynihan P, Watanabe R, Taylor GW et al. (2010) Longitudinal relationship between dietary u-3 fatty acids and periodontal disease. Nutrition 26: 1105–1109. doi:10.1016/j.nut.2009.09.010. PubMed: 20097537. 20097537 10.1016/j.nut.2009.09.010

[30] Naqvi AZ, Buettner C, Phillips RS, Davis RB, Mukamal KJ (2010) n-3 Fatty Acids and Periodontitis in US Adults. J Am Diet Assoc 110: 1669-1675. doi:10.1016/j.jada.2010.08.009. PubMed: 21034880. 21034880 10.1016/j.jada.2010.08.009 PMC3320731

[31] Vardar S, Buduneli N, Buduneli E, Kardeşler L, Baylas H et al. (2006) Dietary Supplementation of Omega-3 Fatty Acid and Circulating Levels of Interleukin-1β, Osteocalcin, and C-Reactive Protein in Rats. J Periodontol 77: 814-820. doi:10.1902/jop.2006.050214. PubMed: 16671873. 10.1902/jop.2006.050214 16671873

[32] Kesavalu L, Bakthavatchalu V, Rahman MM, Su J, Raghu B et al. (2007) Omega-3 fatty acid regulates inflammatory cytokine/mediator messenger RNA expression in *Porphyromonasgingivalis*-induced experimental periodontal disease. Oral Microbiol Immunol 22: 232–239. doi:10.1111/j.1399-302X.2007.00346.x. PubMed: 17600534. 17600534 10.1111/j.1399-302X.2007.00346.x

[33] Bendyk A, Marino V, Zilm PS, Howe P, Bartold PM (2009) Effect of dietary omega-3 polyunsaturated fatty acids on experimental periodontitis in the mouse. J Periodont Res 44: 211–216. doi:10.1111/j.1600-0765.2008.01108.x. PubMed: 19210341. 19210341 10.1111/j.1600-0765.2008.01108.x

[34] Boyle WJ, Simonet WS, Lacey DL (2003) Osteoclast differentiation and activation. Nature 423: 337–342. doi:10.1038/nature01658. PubMed: 12748652. 12748652 10.1038/nature01658

[35] Jules J, Ashley JW, Feng S (2010) Selective Targeting of RANK Signaling Pathways as New Therapeutic Strategies for Osteoporosis. Expert Opin Ther Targets 14: 923–934. doi:10.1517/14728222.2010.511179. PubMed: 20678025. 20678025 10.1517/14728222.2010.511179 PMC2929902

[36] Wada N, Maeda H, Yoshimine Y, Akamine A (2004) Lipopolysaccharide stimulates expression of osteoprotegerin and receptor activator of NF-kappa B ligand in periodontal ligament fibroblasts through the induction of interleukin-1 beta and tumor necrosis factor-alpha. Bone 35: 629-635. doi:10.1016/j.bone.2004.04.023. PubMed: 15336598. 15336598 10.1016/j.bone.2004.04.023

[37] Benatti BB, Silvério KG, Casati MZ, Sallum EA, Nociti FH (2009) Inflammatory and bone-related genes are modulated by aging in human periodontal ligament cells. Cytokine 46: 176–181. doi:10.1016/j.cyto.2009.01.002. PubMed: 19251432. 19251432 10.1016/j.cyto.2009.01.002

[38] Campisi J (2003) Cellular senescence and apoptosis: how cellular responses might influence aging phenotypes. Exp Gerontol 38: 5–11. doi:10.1016/S0531-5565(02)00152-3. PubMed: 12543256. 12543256 10.1016/s0531-5565(02)00152-3

[39] Enoki N, Kiyoshima T, Sakai T, Kobayashi I, Takahashi K et al. (2007) Age-dependent changes in cell proliferation and cell death in the periodontal tissue and the submandibular gland in mice: a comparison with other tissues and organs. J Mol Histol 38: 321–332. doi:10.1007/s10735-007-9105-6. PubMed: 17578672. 17578672 10.1007/s10735-007-9105-6

[40] Jarnbring F, Somogyi E, Dalton J, Gustafsson A, Klinge B (2002) Quantitative assessment of apoptotic and proliferative gingival keratinocytes in oral and sulcular epithelium in patients with gingivitis and periodontitis. J Clin Periodontol 29: 1065–1071. doi:10.1034/j.1600-051X.2002.291203.x. PubMed: 12492905. 12492905 10.1034/j.1600-051x.2002.291203.x

[41] Håkansson J, Eliasson B, Smith U, Enerbäck S (2011) Adipocyte mitochondrial genes and the forkhead factor FOXC2 are decreased in type 2 diabetes patients and normalized in response to rosiglitazone. Diabetol Metab Syndr 3: 32. doi:10.1186/1758-5996-3-32. PubMed: 22098677. 22098677 10.1186/1758-5996-3-32 PMC3230127

[42] Barja G (2007) Mitochondrial oxygen consumption and reactive oxygen species production are independently modulated: implications for aging studies. Rejuvenation Res 10: 215-224. doi:10.1089/rej.2006.0516. PubMed: 17523876. 17523876 10.1089/rej.2006.0516

[43] Gilmer LK, Ansari MA, Roberts KN, Scheff SW (2010) Age-related changes in mitochondrial respiration and oxidative damage in the cerebral cortex of the Fischer 344 rat. Mech Ageing Dev 131: 133-143. doi:10.1016/j.mad.2009.12.011. PubMed: 20080122. 20080122 10.1016/j.mad.2009.12.011 PMC2834189

[44] Santos-González M, López-Miranda J, Pérez-Jiménez F, Navas P, Villalba JM (2012) Dietary oil modifies the plasma proteome during aging in the rat. AGE 34: 341-358. doi:10.1007/s11357-011-9239-z. PubMed: 21472381. 21472381 10.1007/s11357-011-9239-z PMC3312633

